# Network meta-analysis of the efficacy and adverse effects of several treatments for advanced/metastatic prostate cancer

**DOI:** 10.18632/oncotarget.19810

**Published:** 2017-08-02

**Authors:** Jing Wu, Wei-Kang Chen, Wei Zhang, Jin-Song Zhang, Jian-He Liu, Yong-Ming Jiang, Ke-Wei Fang

**Affiliations:** ^1^ Department of Biochemistry and Molecular Biology, School of Basic Medical Sciences, Kunming Medical University, Kunming 650500, P.R. China; ^2^ Department of Urology, The Second Affiliated Hospital of Kunming Medical University, Kunming 650101, P.R. China

**Keywords:** prostate cancer, endocrine therapy, efficacy, adverse effects, randomized controlled trials

## Abstract

This network meta-analysis was conducted to compare the efficacy and adverse effects of several treatments for advanced/metastatic prostate cancer (PC). The PubMed and Cochrane Library databases were searched for randomized controlled trials of treatments for advanced/metastatic PC. Eighteen studies covering 6,340 patients were included in this analysis. The calculated were odds ratios, 95% confidence intervals, and the surface under the cumulative ranking (SUCRA) curve. Pairwise meta-analysis showed that overall survival rates achieved with radiotherapy or endocrine therapy were lower than obtained with radiotherapy + endocrine therapy. The endocrine therapy includes estrogen therapy, luteinizing hormone-releasing hormone agonist (LHRH-A), anti-androgen therapy (ADT), ADT + LHRH-A and estrogen therapy + LHRH-A, and its SUCRA values indicated that for overall response rate, estrogen therapy + LHRH-A ranked the highest (92.6%); for overall survival rate, ADT ranked the highest (75.2%); for anemia, estrogen therapy ranked the highest (88.2%); and for diarrhea and hot flushes, ADT ranked the highest (diarrhea, 87.4%; hot flushes, 89.3%). Cluster analysis on the endocrine therapy showed that ADT + LHRH-A achieved the highest overall survival and overall response rates in the treatment of advanced/metastatic PC. Estrogen therapy and ADT had the lowest incidences of diarrhea and anemia. Thus, combined radiotherapy + endocrine therapy had higher overall survival rate, and among the endocrine therapy, in terms of overall response rate and overall survival rate, ADT + LHRH-A may be a better regimen in the treatment of advanced or metastatic PC.

## INTRODUCTION

In 2008, prostate cancer (PC) is the second most common cause of cancer and the sixth leading cause of cancer death among men worldwide [1]. Often patients present with locally advanced PC, which is a serious condition. In the UK, for example, > 27% of new PC presentations are in locally advanced stage [2]. Diet, physical activity, older age, black race/ethnicity, and a family history of the disease are well-established risk factors for PC [3–5]. Options for treating locally advanced PC include watchful waiting, radiotherapy, and hormone monotherapy or radiation therapy combined with androgen deprivation. Radiotherapy is most commonly used in conjunction with neo-adjuvant, concomitant and/or adjuvant hormone treatment [2]. In this study, we took treatments for advanced/metastatic PC classified as radiotherapy, endocrine therapy and radiotherapy + endocrine therapy. Furthermore, endocrine regimens were also searched including estrogen therapy, luteinizing hormone-releasing hormone agonist (LHRH-A), anti-androgen therapy (ADT), ADT + LHRH-A and estrogen therapy + LHRH-A.

Results from large retrospective studies suggest salvage radiation therapy after biochemical recurrence may be associated with long-term freedom from cancer recurrence [6, 7]. There are studies further suggest that radiotherapy combined with long-term adjuvant ADT is superior to radiotherapy alone for locally advanced PC [8, 9], though data on the efficacy of ADT alone are somewhat limited. In 2012, for example, Mottet et al. confirmed that better progression-free survival, loco-regional control, and metastasis-free survival were achieved with radiotherapy combined with long-term adjuvant ADT than with radiotherapy alone [10]. While in Japan, ADT consisting of an androgen antagonist plus either a LHRH-A or orchiectomy, was the standard care for patients with advanced PC [11, 12]. Usually, there are combined regimens in treating PC, for instance, gonadotropin-releasing hormone agonist commonly used for ADT, needs combination of anti-androgen drugs, because it has risk of flare-up effect at the first dose and testosterone fluctuation [13]. Although numerous studies have been conducted comparing various treatments for advanced/metastatic PC, there has been no comprehensive analysis to compare the efficacies and adverse effects of the three main treatments and the different drugs used for endocrine therapy. We therefore performed a network meta-analysis of overall survival rate, overall response rates, anemia, diarrhea and hot flushes. The advantage of network meta-analysis over standard pairwise meta-analysis is that it allows for indirect comparisons, more data are incorporated in the analysis, and the bigger picture is tackled, so that the results of comparison were more reliable [14]. Our aim was to compare the efficacy and adverse effects of radiotherapy, endocrine therapy and radiotherapy + endocrine therapy for the treatment of advanced/metastatic PC. We anticipate our findings will be helpful for surgeons planning treatment strategies for these patients.

## RESULTS

### Baseline characteristics of included studies

A total of 3,361 relevant studies were initially retrieved. We first excluded 125 duplicate studies, 541 letters and reviews or meta-analyses, 89 non-human studies, and 292 non-English studies. After full-text review, of the remaining 2,314 studies, 732 non-randomized controlled trials, 993 unrelated to advanced/metastatic PC, 569 unrelated to treatments and 2 without data integrity or with no data were further ruled out. Ultimately, 18 randomized controlled trials published between 1988 and 2017 were eligible for this meta-analysis [10, 15–31] (Supplementary Figure 1). These studies included 6,340 patients with advanced/metastatic PC, aged from 40 to 90, most of whom were treated with LHRH-A or ADT + LHRH-A. There were 16 studies in Caucasians and 2 in Asians, and all 18 included studies were two-arm trials. The baseline characteristics of included studies are listed in Supplementary Table 1.

### Pairwise meta-analysis of the different treatments of advanced/metastatic PC

**Figure 1:**
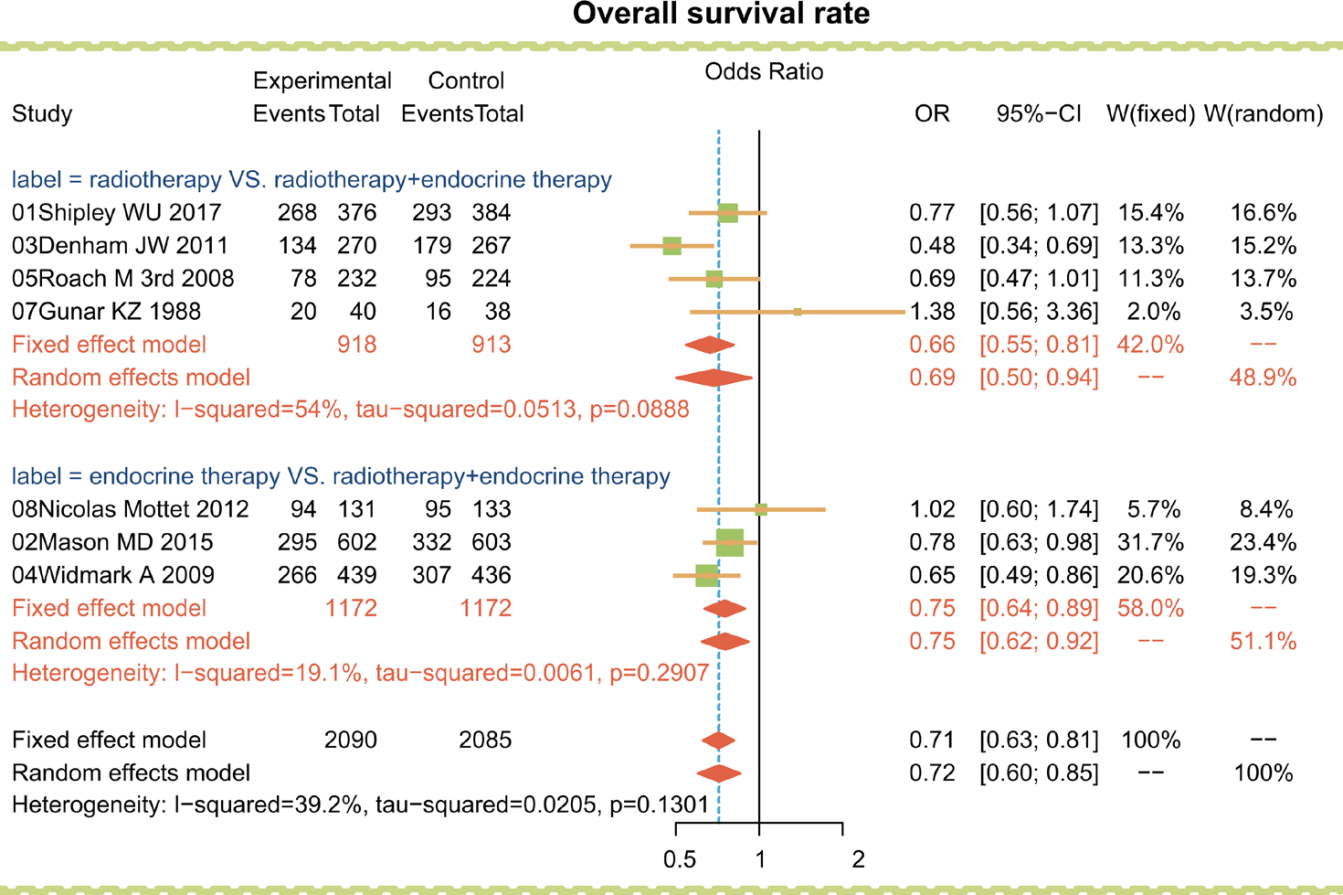
Traditional forest plots for overall survival rate among advanced/metastatic PC patients treated with radiotherapy, endocrine therapy and radiotherapy + endocrine therapy

Firstly, radiotherapy, endocrine therapy and radiotherapy + endocrine therapy were compared directly, and the results as shown in Figure 1, radiotherapy and endocrine therapy each had lower overall survival rates than radiotherapy + endocrine therapy (OR = 0.69, 95% CI = 0.50 ~ 0.94; OR = 0.75, 95% CI = 0.64 ~ 0.89, respectively). It thus appears combined radiotherapy + endocrine therapy significantly improved the overall survival rate in the treatment of advanced/metastatic PC than either radiotherapy or endocrine therapy alone.

Then, the endocrine regimens including estrogen therapy, LHRH-A, ADT, ADT + LHRH-A and estrogen therapy + LHRH-A were compared directly. As shown in Table 1, the overall survival rate was lower among patients treated with LHRH-A than among those treated with ADT + LHRH-A (OR = 0.65, 95% CI = 0.48 ~ 0.87). However, in terms of overall response rate, there was no significant difference among the endocrine therapies. In terms of the adverse event, LHRH-A had higher rates of anemia and hot flushes than ADT (OR = 5.00, 95% CI = 1.49 ~ 16.83; OR = 287.5, 95% CI = 24.41 ~ 3386.56, respectively). ADT had lower rates of diarrhea and hot flushes than ADT + LHRH-A (OR = 0.10, 95% CI = 0.02 ~ 0.44; OR = 0.35, 95% CI = 0.20 ~ 0.61, respectively), while LHRH-A had a lower diarrhea rate than ADT + LHRH-A (OR = 0.30, 95% CI = 0.16 ~ 0.56). Estrogen therapy had a lower rate of hot flushes than LHRH-A (OR = 0.28, 95% CI = 0.17 ~ 0.47). In sum, ADT + LHRH-A had a higher overall survival rate than LHRH-A but also higher rate of adverse events than ADT or LHRH-A alone.

**Table 1:** Pairwise meta-analysis for efficacy or adverse events in advanced or metastatic prostate cancer

Included studies	Comparisons	Efficacy/Adverse events		Pairwise meta-analysis
		Treatment1	Treatment2	OR (95% CI)
**Overall response rate**				
2 studies	estrogen therapy vs. LHRH-A	77/143	97/172	0.79 (0.31–2.01)
2 studies	estrogen therapy vs. ADT	21/137	16/131	1.15 (0.48–2.75)
1 study	LHRH-A vs. ADT + LHRH-A	19/62	24/63	0.72 (0.34–1.51)
1 study	ADT + LHRH-A vs. estrogen therapy + LHRH-A	12/22	22/29	0.38 (0.12–1.26)
**Overall survival rate**				
1 study	estrogen therapy vs. LHRH-A	43/124	40/124	1.11 (0.66–1.89)
1 study	estrogen therapy vs. ADT	53/102	50/101	1.10 (0.64–1.91)
2 studies	LHRH-A vs. ADT + LHRH-A	203/401	243/405	**0.65 (0.48–0.87)**
1 study	ADT vs. ADT + LHRH-A	65/108	66/112	1.05 (0.61–1.81)
1 study	ADT + LHRH-A vs. estrogen therapy + LHRH-A	11/22	14/29	1.07 (0.35–3.25)
**Anemia**				
1 study	estrogen therapy vs. ADT	27/104	33/109	0.81 (0.44–1.47)
1 study	LHRH-A vs. ADT + LHRH-A	7/101	11/102	0.62 (0.23–1.66)
1 study	LHRH-A vs. ADT	20/26	10/25	**5.00 (1.49–16.83)**
1 study	ADT+LHRH-A vs. estrogen therapy + LHRH-A	2/22	4/29	0.62 (0.10–3.77)
**Diarrhea**				
2 studies	estrogen therapy vs. ADT	34/146	27/143	1.32 (0.74–2.35)
2 studies	LHRH-A vs. ADT + LHRH-A	13/330	43/327	**0.30 (0.16–0.56)**
1 study	ADT vs. ADT + LHRH-A	2/108	18/112	**0.10 (0.02–0.44)**
1 study	ADT + LHRH-A vs. estrogen therapy + LHRH-A	0/22	1/29	0.42 (0.02–10.87)
**Hot flushes**				
3 studies	estrogen therapy vs. LHRH-A	40/278	75/249	**0.28 (0.17–0.47)**
1 study	estrogen therapy vs. ADT	0/42	2/34	0.15 (0.01–3.30)
1 study	LHRH-A vs. ADT	25/26	2/25	**287.50 (24.41–3386.56)**
2 studies	LHRH-A vs. ADT + LHRH-A	197/369	186/366	1.39 (0.53–3.63)
1 study	ADT vs. ADT + LHRH-A	40/108	70/112	**0.35 (0.20–0.61)**

Notes: OR = odd ratio; 95% CI = 95% confidence intervals; LHRH-A = luteinizing hormone-releasing hormone agonist; ADT = anti-androgen therapy. Bolded numbers represent the differences are of significance.

### Network meta-analysis of the different endocrine therapies for advanced/metastatic PC

**Figure 2:**
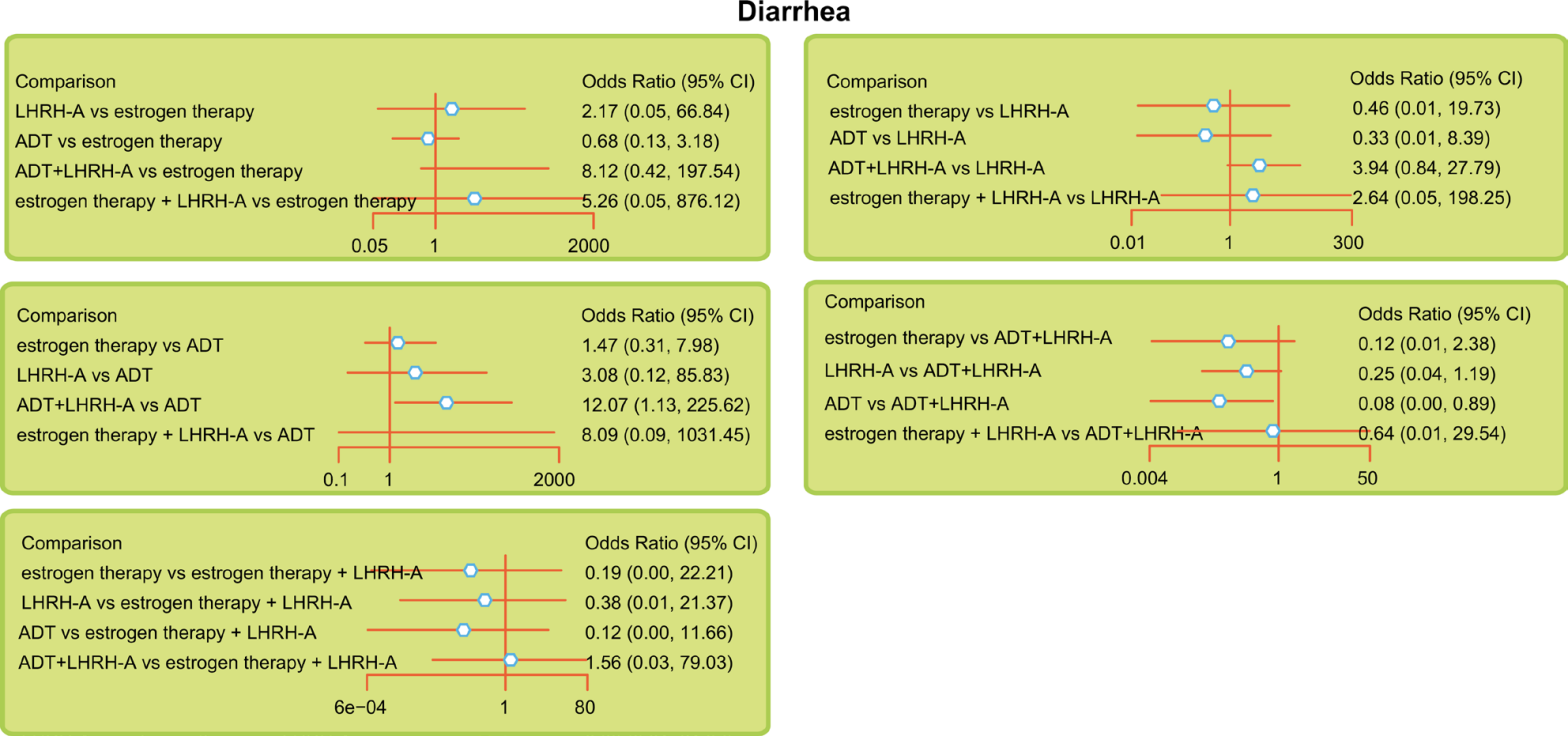
Relative forest plots for diarrhea during the indicated treatments of advanced/metastatic PC Note: LHRH-A = luteinizing hormone-releasing hormone agonist; ADT = anti-androgen therapy.

The five endocrine regimens were also made an indirect comparison, and the results of this network meta-analysis indicated that compared with ADT + LHRH-A, ADT had a lower rate of diarrhea (OR = 0.08, 95% CI = 0.00 ~ 0.89). However, in terms of the overall response rate, overall survival rate, and the anemia and hot flush rates, there was no significant difference among the endocrine therapies used in the treatment of advanced/metastatic PC (Table 2 and Figure 2).

**Table 2:** Odd ratios and 95% confidence intervals of five treatment modalities of five endpoint outcomes

Odd ratios (95% confidence intervals)				
**Overall response rate**				
estrogen therapy	1.34 (0.44, 3.35)	0.78 (0.22, 2.97)	1.91 (0.32, 9.07)	5.35 (0.46, 50.09)
0.74 (0.30, 2.26)	LHRH-A	0.58 (0.13, 3.23)	1.41 (0.36, 5.35)	3.89 (0.49, 31.61)
1.28 (0.34, 4.51)	1.72 (0.31, 7.84)	ADT	2.48 (0.26, 17.30)	6.75 (0.43, 82.70)
0.52 (0.11, 3.12)	0.71 (0.19, 2.76)	0.40 (0.06, 3.80)	ADT + LHRH-A	2.78 (0.55, 13.93)
0.19 (0.02, 2.15)	0.26 (0.03, 2.04)	0.15 (0.01, 2.34)	0.36 (0.07, 1.81)	estrogen therapy + LHRH-A
**Overall survival rate**				
estrogen therapy	0.90 (0.45, 1.83)	1.44 (0.47, 4.47)	1.40 (0.57, 3.24)	1.27 (0.29, 5.61)
1.11 (0.55, 2.24)	LHRH-A	1.61 (0.67, 3.94)	1.53 (0.94, 2.51)	1.41 (0.40, 5.20)
0.69 (0.22, 2.15)	0.62 (0.25, 1.48)	ADT	0.95 (0.46, 1.96)	0.88 (0.21, 3.71)
0.72 (0.31, 1.75)	0.65 (0.40, 1.07)	1.05 (0.51, 2.17)	ADT + LHRH-A	0.92 (0.27, 3.14)
0.79 (0.18, 3.44)	0.71 (0.19, 2.48)	1.13 (0.27, 4.74)	1.09 (0.32, 3.65)	estrogen therapy + LHRH-A
**Anemia**				
estrogen therapy	6.63 (0.33, 129.95)	1.26 (0.17, 8.64)	10.57 (0.29, 447.21)	20.03 (0.21, 2021.57)
0.15 (0.01, 3.07)	LHRH-A	0.19 (0.02, 1.72)	1.67 (0.20, 15.04)	3.15 (0.10, 98.48)
0.79 (0.12, 5.99)	5.37 (0.58, 55.14)	ADT	8.55 (0.42, 225.47)	15.71 (0.29, 1070.30)
0.09 (0.00, 3.48)	0.60 (0.07, 5.01)	0.12 (0.00, 2.39)	ADT + LHRH-A	1.80 (0.12, 31.43)
0.05 (0.00, 4.87)	0.32 (0.01, 10.25)	0.06 (0.00, 3.50)	0.56 (0.03, 8.68)	estrogen therapy + LHRH-A
**Diarrhea**				
estrogen therapy	2.17 (0.05, 66.84)	0.68 (0.13, 3.18)	8.12 (0.42, 197.54)	5.26 (0.05, 876.12)
0.46 (0.01, 19.73)	LHRH-A	0.33 (0.01, 8.39)	3.94 (0.84, 27.79)	2.64 (0.05, 198.25)
1.47 (0.31, 7.98)	3.08 (0.12, 85.83)	ADT	**12.07 (1.13, 225.62)**	8.09 (0.09, 1031.45)
0.12 (0.01, 2.38)	0.25 (0.04, 1.19)	**0.08 (0.00, 0.89)**	ADT + LHRH-A	0.64 (0.01, 29.54)
0.19 (0.00, 22.21)	0.38 (0.01, 21.37)	0.12 (0.00, 11.66)	1.56 (0.03, 79.03)	estrogen therapy + LHRH-A
**Hot flushes**				
estrogen therapy	7.67 (0.59, 154.46)	0.43 (0.01, 19.24)	3.26 (0.07, 195.76)	
0.13 (0.01, 1.70)	LHRH-A	0.06 (0.00, 1.64)	0.42 (0.02, 8.68)	
2.35 (0.05, 95.33)	17.94 (0.61, 651.76)	ADT	7.83 (0.20, 340.12)	
0.31 (0.01, 14.53)	2.37 (0.12, 49.49)	0.13 (0.00, 4.97)	ADT + LHRH-A	

Notes: Odd ratios and 95% confidence intervals below the treatments should be read from row to column while above the treatments should be read from column to row. LHRH-A = luteinizing hormone-releasing hormone agonist; ADT = anti-androgen therapy. Bolded numbers represent the differences are of significance.

### SUCRA values for efficacy and adverse effects in the treatment of advanced/metastatic PC

SUCRA values of the five endocrine regimens showed in terms of overall response rate, estrogen therapy + LHRH-A had the highest SUCRA value (92.6%); in terms of overall survival rate, ADT had the highest SUCRA value (75.2%) (Table 3). Considering adverse events in terms of anemia, estrogen therapy had the highest SUCRA value (88.2%); in terms of diarrhea and hot flush rates, ADT had the highest SUCRA value (diarrhea: 87.4%; hot flushes: 89.3%). We therefore conclude that in the treatment of advanced/metastatic PC, ADT and estrogen therapy + LHRH-A significantly improved the overall survival rate and overall response rate respectively, while estrogen therapy decreased the anemia rate and ADT decreased the diarrhea and hot flush rates significantly.

**Table 3:** SUCRA values of five treatment modalities under five endpoint outcomes

Treatments	SUCRA values (%)				
	Overall response rate	Overall survival rate	Anemia	Diarrhea	Hot flushes
estrogen therapy	43.8	48.6	**88.2**	73.0	75.5
LHRH-A	57.2	37.0	53.0	63.4	32.8
ADT	37.6	**75.2**	82.8	**87.4**	**89.3**
ADT + LHRH-A	68.6	74.8	41.8	30.4	52.5
estrogen therapy + LHRH-A	**92.6**	64.4	33.2	45.0	NR

Notes: SUCRA = surface under the cumulative ranking curves; NR = not report; LHRH-A = luteinizing hormone-releasing hormone agonist; ADT = anti-androgen therapy. Bold numbers represent the SUCRA is higher compared to the other interventions.

### Cluster analysis of different treatments for advanced/metastatic PC

**Figure 3:**
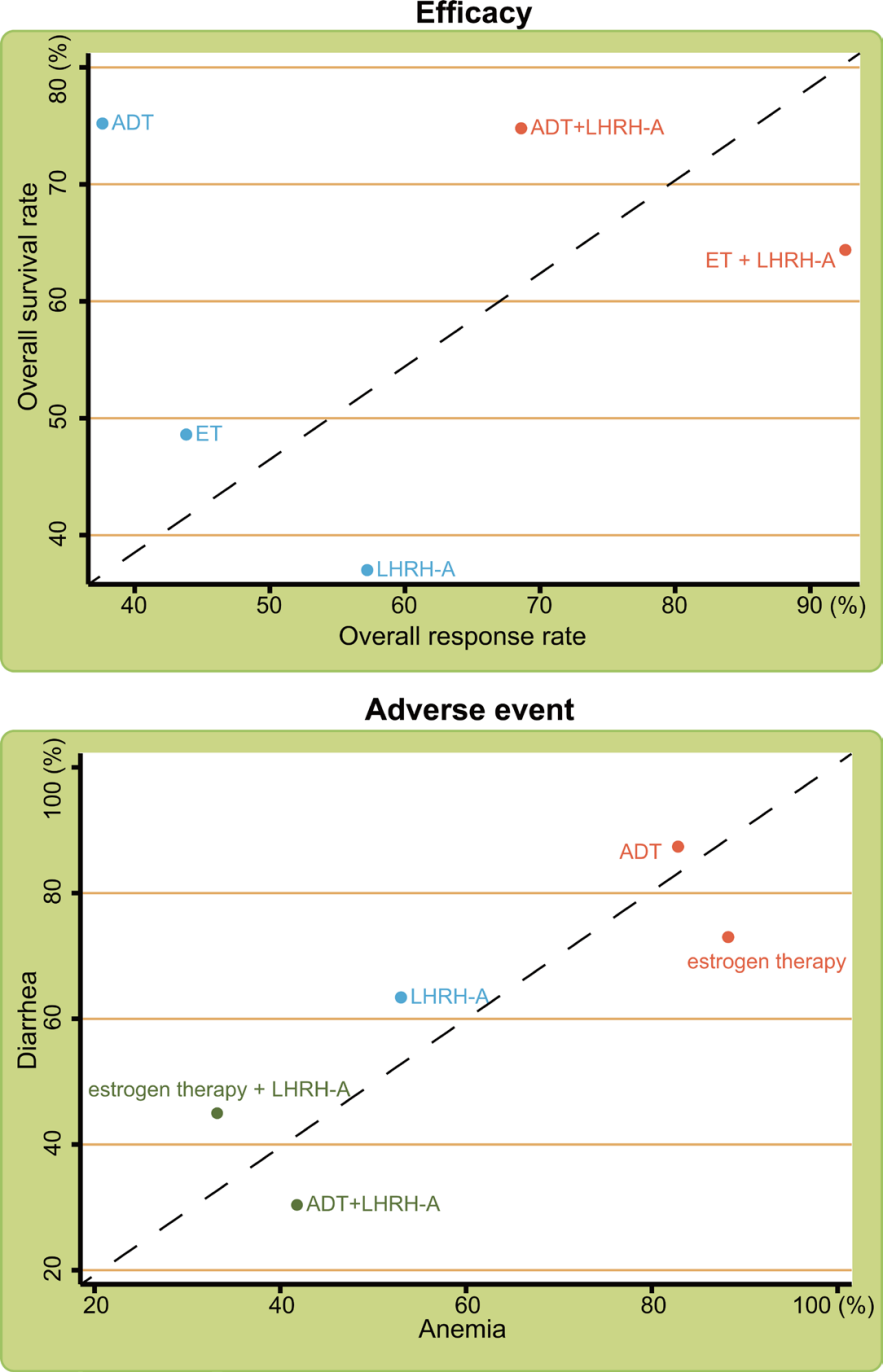
Cluster analyses of the efficacy and adverse events of the indicated treatments for advanced/metastatic PC Note: LHRH-A = luteinizing hormone-releasing hormone agonist; ADT = anti-androgen therapy.

Consistent with the SUCRA values, cluster analysis on the five endocrine regimens showed that ADT + LHRH-A and estrogen therapy + LHRH-A improved the overall survival rate and overall response rate in the treatment of advanced/metastatic PC, while estrogen therapy and ADT had the lowest rates of diarrhea and hot flushes (Figure 3). However, because the results of estrogen therapy + LHRH-A are based on small sample sizes (*n* = 29), further confirmation is needed.

### Publication bias analysis

Cochrane system bias evaluation is shown in Supplementary Figure 2. All the included studies clearly satisfied criteria for adequate sequence generation, allocation concealment, and blinding, and 50% clearly met the criteria for incomplete outcome data addressed, free of selective reporting, and free of other bias. Nearly 30% was uncertain, and only about 10% did not meet the criteria. The quality of the included studies was generally high, and the publication bias was small. The funnel plot is graphically symmetrical, suggesting no obvious publication bias (Supplementary Figure 3).

## DISCUSSION

This study shows that compared to radiotherapy and endocrine therapy, alone, radiotherapy + endocrine therapy was most efficacious in the treatment of advanced/metastatic PC. Salvage radiotherapy is often necessary in men who have undergone radical prostatectomy but show evidence of PC recurrence signaled by persistently or recurrently elevated prostate-specific antigen (PSA) levels [15]. For such patients, several large randomized studies indicate that combining radiotherapy and androgen-deprivation improves outcome over radiotherapy alone. The survival benefit depends on the duration of the hormone treatment [32, 33]. In that respect breast cancer and PC are analogous: in both disorders, long-term outcome is improved by the combination of radiotherapy + endocrine therapy [34, 35], and long-term adjuvant endocrine therapy significantly improves overall survival [36].

Pairwise meta-analysis showed that a higher overall survival rate was achieved with ADT + LHRH-A than with LHRH-A, but both LHRH-A and ADT + LHRH-A had higher rates of anemia, diarrhea and hot flushes than ADT. Anemia is common in men with advanced PC, which may be caused by several factors, including nutritional decline, androgen deprivation, treatment-related toxicity, bone marrow infiltration, and the chronic inflammatory state [37]. Hot flashes and bone mineral loss commonly occur with LHRH-A while estrogen helps prevent these side-effects [38]. When PC patients receiving ADT, side effects such as changes in lipid profiles, osteoporosis, and anemia may have significant morbidity, while other side effects such as impotence, decreased libido, fatigue, and hot flashes primarily affect the patient's quality of life [39]. Sexual dysfunction, hot flashes and osteoporosis were reported in the combined treatment of ADT + LHRH-A [40]. For instance, bicalutamide is a nonsteroidal androgen antagonist that competitively inhibits the action of androgens by binding to androgen receptors in the target tissues [41]. Patients receiving bicalutamide (80 mg) combination therapy are significantly more likely to achieve (prostate-specific antigen) PSA levels ≤ 4 ng/ml and have higher overall tumor-response rates than those receiving LHRH-A alone [42]. The tolerability profile of bicalutamide makes it an attractive agent for use in hormone combination regimens, particularly as the profile is more favorable than other anti-androgens [43, 44].

The SUCRA results revealed that ADT and estrogen therapy + LHRH-A had higher overall survival rate in the treatment of advanced/metastatic PC, but estrogen therapy and ADT had lower a rate of anemia, diarrhea and hot flushes. In addition, although short-term neo-adjuvant androgen suppression followed by radical prostatectomy was associated with a lower rate of positive margins and lower tumor pathological stage, there was no difference in overall survival [45, 46]. Because low bone mineral density independently predicts fracture risk, men receiving bicalutamide monotherapy may have a lower risk of fracture than those receiving a gonadotropin-releasing hormone agonist [47, 48]. Androgen and estrogen receptors are expressed in osteoblasts and osteoclasts [49, 50], and their ligands contribute to the regulation of both bone formation and bone resorption in men [51, 52]. In two randomized controlled trials, pamidronate prevented bone loss in men treated with a gonadotropin-releasing hormone agonist [53, 54]. Furthermore, we made a cluster analysis on the different endocrine therapies. The results showed that ADT + LHRH-A and estrogen therapy + LHRH-A had higher overall response rate and overall survival rate. However, small sample sizes of estrogen therapy + LHRH-A could not give a confirm conclusion that estrogen therapy + LHRH-A had better efficacy in treating PC. Thus, to ensure the preciseness of this study, we finally get a conclusion that ADT + LHRH-A had higher overall response rate and overall survival rate in treating advanced/metastatic PC.

There are several limitations to our study. First, there were differences in the numbers of participants in the pairwise comparisons among the interventions, and the number of the included studies was relatively small, which may have impacted the results. Second, the reported clinical characteristics were incomplete in some of the included studies. For example, some studies did not provide information related the PSA or Gleason score. Consequently, we could not conduct a meta-regression analysis. Nonetheless, the advantage of our study is that we comprehensively compared the efficacies and adverse events of the three main treatments of advanced/metastatic PC: radiotherapy, endocrine therapy (estrogen therapy, LHRH-A, ADT, ADT + LHRH-A and estrogen therapy + LHRH-A) and radiotherapy + estrogen therapy. Based on our results, we concluded that combined radiotherapy + endocrine therapy may be the best treatment for advanced/metastatic PC, and that ADT and estrogen therapy + LHRH-A had the greatest efficacy in the treatment of advanced/metastatic PC. However, there is a need for further study of these treatments so that they will continue to evolve, and the treatment of advanced/metastatic PC will continue to improve.

## MATERIALS AND METHODS

### Search strategy

PubMed, the Cochrane library and other English language databases were searched from the inception of each database to February 2017. Searches were conducted using combinations of keywords and free words: prostate cancer, radiotherapy, endocrine therapy, anti-androgen therapy, estrogen therapy, luteinizing hormone-releasing hormone (LHRH) agonist and randomized controlled trials, among others.

### Inclusion and exclusion criteria

The inclusion criteria were: (1) study design: randomized controlled trials; (2) interventions: radiotherapy, endocrine therapy and radiotherapy + endocrine therapy; among and estrogen therapies were estrogen, LHRH-A, ADT, ADT + LHRH-A and estrogen + LHRH-A; (3) study subjects: patients with advanced or metastatic PC; (4) outcomes: efficacy (overall response rate; overall survival rate) and adverse events (anemia; diarrhea; hot flushes). The exclusion criteria were: (1) patients started treatment before randomization; (2) patients with significant inter-current medical conditions, prior malignancies or metastases within the previous 5 years; (3) candidates for watchful waiting; (4) studies with insufficient data integrity (e.g., non-paired studies); (5) non-randomized controlled trials; (6) repeated published literature; (7) conference reports, systematic reviews or summaries; (8) non-human studies and non-English studies.

### Data extraction and quality assessment

The search for suitable literature, extraction of data and assessment of study quality was performed independently by two investigators, and disagreements were resolved by consensus. The risk of bias of the included studies was assessed by more than two investigators using the CCRBT (Cochrane Collaboration risk of bias tool) [55]. The CCRBT assesses the quality of randomized controlled trials using six domains: sequence generation, allocation concealment, blinding, incomplete outcome data, selective outcome reporting, and other potential threats to validity. The assessment is scored “yes”, “no”, or “unclear”, respectively, indicating a low, high, or unclear risk of bias. If one or no domain was identified as “unclear” or “no,” the study was classified as having a low risk of bias. If two or three domains were deemed “unclear” or “no”, the study is classified as having a moderate risk of bias. If four or more domains were deemed “unclear” or “no”, the study is classified as having a high risk of bias [56]. Review Manager 5 (RevMan 5.2.3, Cochrane Collaboration, Oxford, UK) was applied to evaluate the quality assessment and investigate publication bias.

### Statistical analysis

Firstly, we performed a pairwise meta-analysis of direct evidence from the included studies using R version 3.2.1 and the meta package. The clinical outcomes of the three treatment protocols for advanced/metastatic PC were estimated using pooled odd ratios (ORs) and 95% confidence intervals (CIs). We used the Chi-square test and I-square test to quantify heterogeneity among studies [57]. Secondly, Bayesian network meta-analyses were performed to compare different interventions with each other. Each analysis was on the basis of non-informative priors for precision and effect sizes. All data sets were analyzed with WinBUGS version in batch mode using the R package. Lack of autocorrelation and convergence were checked and confirmed using four chains and a 20,000-simulation burn-in phase. Direct probability statements were derived from an additional 50,000-simulation phase [58]. Thirdly, with the R 3.2.1 software, two-category data was produced by using metabin function. With OR as effect size, Mantel-Haenszel method was used to calculate fixed effect model or random effect model. When *P* > 0.05, we chose fixed effect model otherwise we chose the random effect model. This meta-analysis was based on the Egger's test, with the metabias function to build General funnel plot. Finally, the surface under the cumulative ranking curve (SUCRA) was used to calculate the probability of each intervention being the most effective treatment based on a Bayesian approach using probability values: the larger the SUCRA value, the better the rank of the intervention [59, 60]. Under the indicators of efficacy and adverse events, we firstly ranked the five endocrine regimens and calculated the rates (Ranking is to sort the five endocrine regimens), and then drew a curve with ranking as abscissa and rate as ordinate, finally we calculate the area under the curve, the calculation formula is = (j = regimen; b = ranking; a = five endocrine regimens; = cumulative rate of j/b). Cluster analyses were adopted to assess the value of five endocrine regimens for advanced/metastatic PC. The treatments were clustered based on the similarity of two variables and yielded the advantages and disadvantages of different treatments [59]. The results of cluster analyses show in Figure 3 where nodes with the same color are in a cluster, and the advantages of the endocrine regimens are in descending order from the upper right to lower left. R version 3.2.1, gemtc version 0.6, and Markov Chain Monte Carlo engine Open BUGS version 3.4.0 were used in all computations.

## SUPPLEMENTARY MATERIALS FIGURES AND TABLES


