# Supplementary material for: Network meta-analysis of the efficacy and adverse effects of several treatments for advanced/metastatic prostate cancer
Source: Oncotarget. 2017 Aug 2;8(35):59709–19. doi: 10.18632/oncotarget.19810 (PMC5601770; doi:10.18632/oncotarget.19810)
Supplement: Supplementary file 1 [file oncotarget-08-59709-s001.pdf]

Network meta-analysis of the efficacy and adverse effects of several treatments for advanced/metastatic prostate cancer

SUPPLEMENTARY MATERIALS

Supplementary Table 1: The baseline characteristics for included studies

First author	Year	Country	Interventions		Total	Sample size		Age (years)		Gleason score		PSA (ng/ml)	
			T1	T2		T1	T2	T1	T2	T1	T2	T1	T2
Shipley WU	2017	America	RT	RT + ET(ADT)	760	376	384	NR	NR	2–10	2–10	NR	NR
Mason MD	2015	England	ET(ADT)	RT + ET(ADT)	1205	602	603	69.7 ± 5.2	69.7 ± 5.4	NR	NR	NR	NR
Nicolas Mottet	2012	France	ET(ADT)	RT + ET(ADT)	264	131	133	71 ± 4.1	72 ± 4.1	4–10	4–10	51.8 (129.3)	41.5 (45.9)
Denham JW	2011	Australia	RT	RT + ET(ADT)	535	270	267	67 (51–80)	68 (41–87)	2–10	2–10	16.4 (0.6–165.0)	14.4(0.5–154.2)
Widmark A	2009	Sweden	ET(LHRH-A + ADT)	RT + ET(LHRH-A + ADT)	875	439	436	66.2 ± 5.1	65.7 ± 5.5	NR	NR	16·0 (8.9–27.0)	16·0 (9.0–26.7)
Roach M 3rd	2008	America	RT	RT + ET(ADT)	456	232	224	71 ± 6.6	70 ± 9.2	3–10	3–10	33.8 (1.9–264.6)	22.6(2.2–128)
Gunar KZ	1988	America	RT	RT + ET(estrogen therapy)	78	40	38	NR	NR	NR	NR	NR	NR
Langley RE	2013	England	estrogen therapy	LHRH-A	254	169	85	73 ± 2.6	75 ± 2.6	4–10	4–10	55 (21–153)	36 (19–106)
Usami M	2007	Japan	LHRH-A	ADT + LHRH-A	203	101	102	NR	NR	NR	NR	NR	NR
Smith MR	2004	America	ADT	LHRH-A	51	25	26	63 ± 8	65 ± 10	NR	NR	40 ± 119	158 ± 670
Noguchi M	2004	Japan	ADT + LHRH-A	estrogen therapy + LHRH-A	57	28	29	NR	NR	2–10	2–10	NR	NR
Boccardo F	1999	Italy	ADT	ADT + LHRH-A	220	108	112	74 ± 7.7	74 ± 7.1	NR	NR	56.7 (0.64–2960.0)	80.3(1.07–6641.0)
Ferrari P	1996	Italy	LHRH-A	ADT + LHRH-A	150	76	74	71.6	68.7	NR	NR	NR	NR
Chang A	1996	France	estrogen therapy	ADT	92	48	44	67 ± 7.1	68 ± 6.1	NR	NR	NR	NR
Waymont B	1992	England	estrogen therapy	LHRH-A	250	126	124	72.6 ± 10.5	72.4 ± 7.2	NR	NR	NR	NR
Citrin DL	1991	America	estrogen therapy	LHRH-A	67	19	48	68–83	70–87	NR	NR	NR	NR
Crawford ED	1989	America	LHRH-A	ADT + LHRH-A	603	300	303	68 ± 15.3	69 ± 8.7	NR	NR	NR	NR
de Kernion JN	1988	America	estrogen therapy	ADT	220	109	111	NR	NR	NR	NR	NR	NR

Note: T = treatment; NR = not report; PSA = prostate specific antigen; RT = radiotherapy; ET = endocrine therapy; LHRH–A = luteinizing hormone releasing hormone; ADT = anti-androgen therapy.

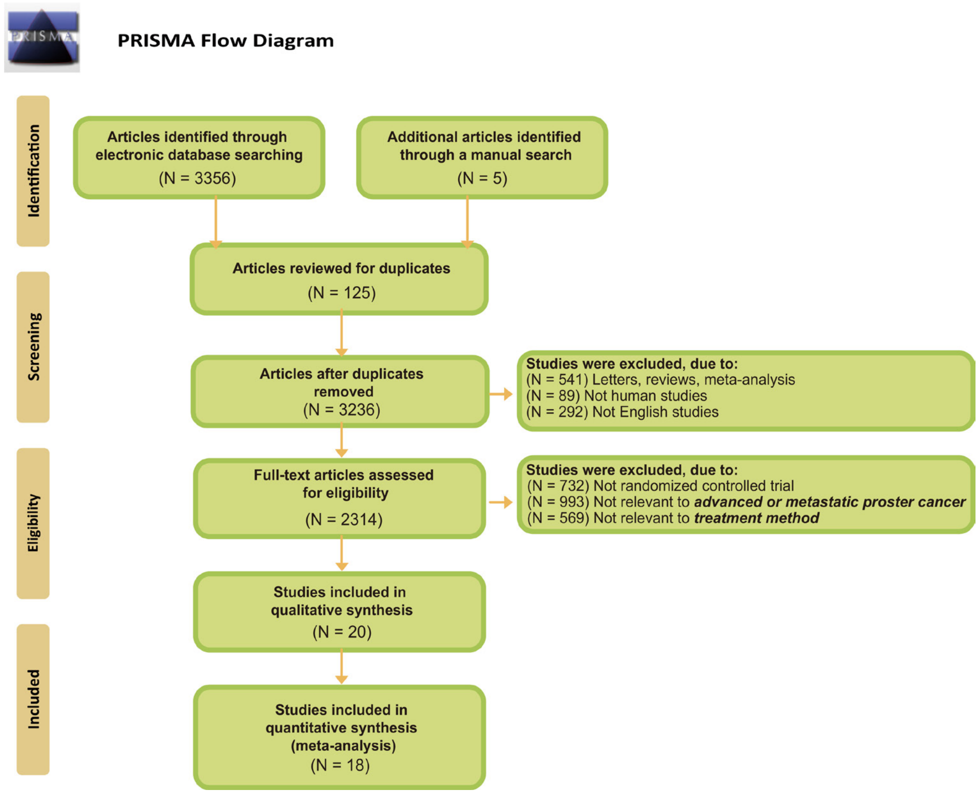

Supplementary Figure 1: Flowchart for the literature search and study selection.

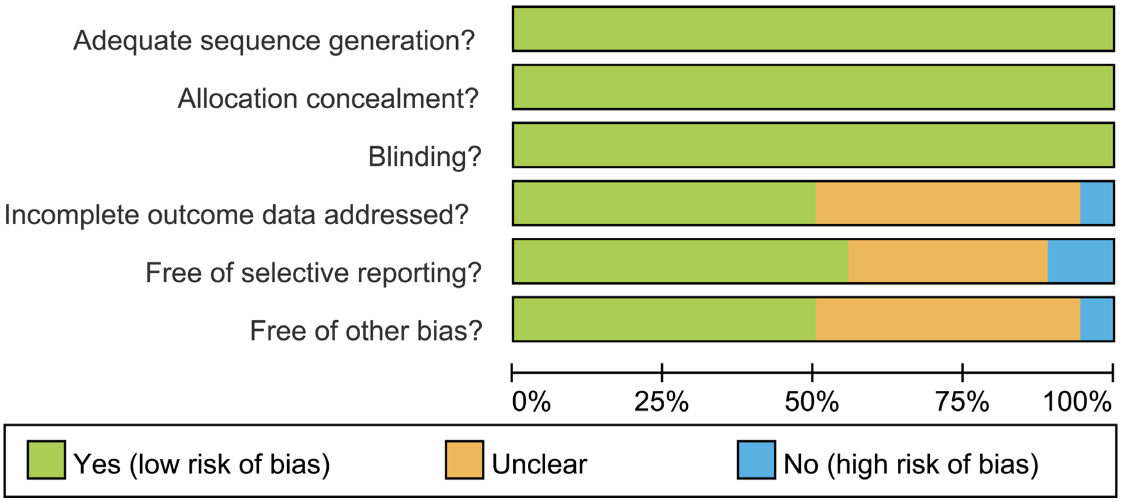

Supplementary Figure 2: Risk of bias in the 18 enrolled studies according to the Cochrane collaboration risk of bias tool.

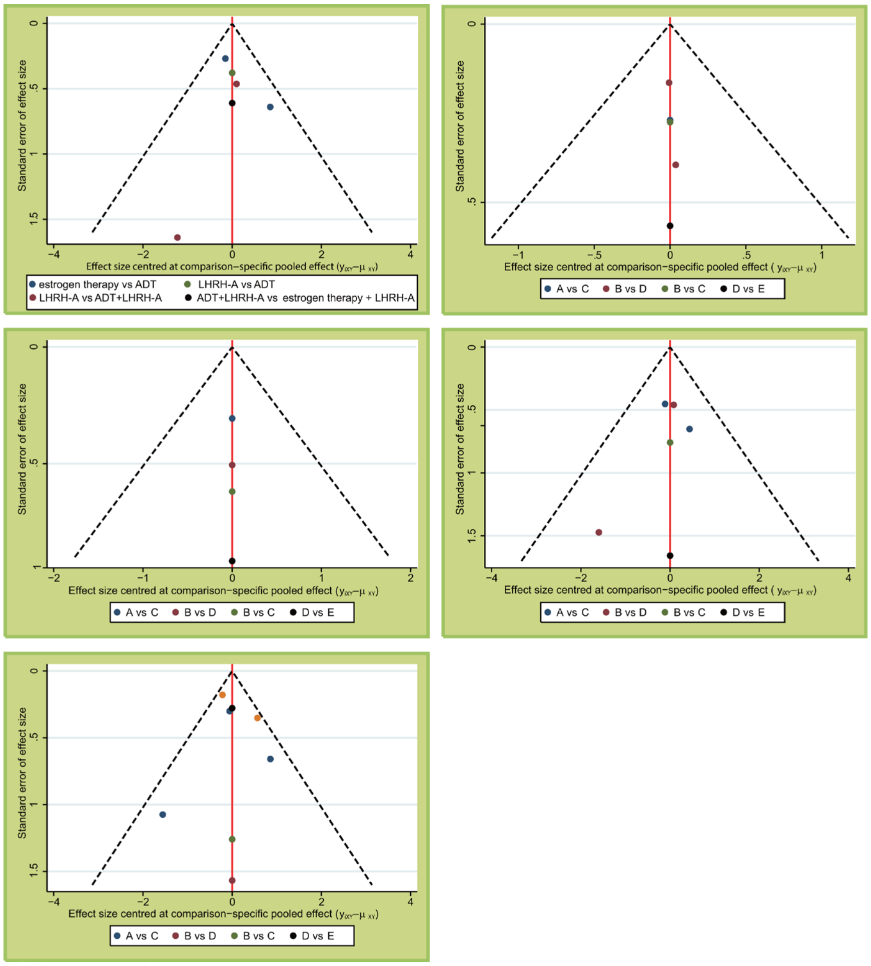

Supplementary Figure 3: Funnel plot for the publication bias analysis of all the included studies. Note: LHRH-A = luteinizing hormone-releasing hormone agonist; ADT = anti-androgen therapy.
